# Organ Fibrosis and Autoimmunity: The Role of Inflammation in TGFβ-Dependent EMT

**DOI:** 10.3390/biom11020310

**Published:** 2021-02-18

**Authors:** Margherita Sisto, Domenico Ribatti, Sabrina Lisi

**Affiliations:** Department of Basic Medical Sciences, Neurosciences and Sensory Organs (SMBNOS), Section of Human Anatomy and Histology, University of Bari “Aldo Moro”, 70124 Bari, Italy; domenico.ribatti@uniba.it (D.R.); sabrina.lisi@uniba.it (S.L.)

**Keywords:** epithelial-mesenchymal transition, fibrosis, TGF-β, autoimmune diseases, inflammation

## Abstract

Recent advances in our understanding of the molecular pathways that control the link of inflammation with organ fibrosis and autoimmune diseases point to the epithelial to mesenchymal transition (EMT) as the common association in the progression of these diseases characterized by an intense inflammatory response. EMT, a process in which epithelial cells are gradually transformed to mesenchymal cells, is a major contributor to the pathogenesis of fibrosis. Importantly, the chronic inflammatory microenvironment has emerged as a decisive factor in the induction of pathological EMT. Transforming growth factor-β (TGF-β), a multifunctional cytokine, plays a crucial role in the induction of fibrosis, often associated with chronic phases of inflammatory diseases, contributing to marked fibrotic changes that severely impair normal tissue architecture and function. The understanding of molecular mechanisms underlying EMT-dependent fibrosis has both a basic and a translational relevance, since it may be useful to design therapies aimed at counteracting organ deterioration and failure. To this end, we reviewed the recent literature to better elucidate the molecular response to inflammatory/fibrogenic signals in autoimmune diseases in order to further the specific regulation of EMT-dependent fibrosis in more targeted therapies.

## 1. Introduction

**Figure 1:**
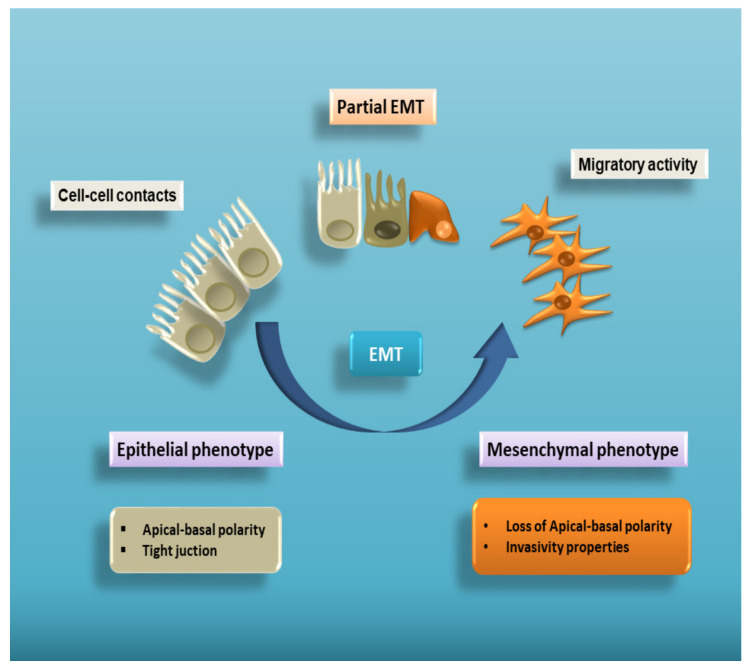
Cell plasticity in EMT. EMT is a multistep process allowing epithelial cells to lose their apical–basal polarity and disassemble epithelial cell–cell contacts. Through a complex cellular and molecular program, these cells progressively acquire mesenchymal features that include cytoskeleton reorganization and a proteolytic capacity favouring cell motility. Therefore, EMT proceeds through multiple partial intermediate states, collectively known as the partial EMT. A partial EMT takes place physiologically during wound healing and leads to an intermediate phenotype that presents some epithelial features, but also characteristics of mesenchymal cells.

Epithelial–mesenchymal transition (EMT) is a complex cellular program known to be a crucial driver in the context of embryonic development, wound healing and tumour progression. EMT involves intense changes in the morphology and behaviour of epithelial cells. Indeed, not only do they lose their original phenotypic and functional features, but they acquire the capacity to degrade the basement membrane, migrating through the extracellular matrix to populate different territories either during embryonic development or cancer progression, or to adopt a profibrotic myofibroblast nature in the tissue interstitial space [1,2,3,4,5]. This process is characterized by a dramatic change in the expression of specific epithelial proteins, such as E-cadherin and zonula occludens-1 (ZO-1), followed by a markedly increased expression of some mesenchymal markers, including α-smooth muscle actin (α-SMA), vimentin, and laminin [6] (Figure 1).

Although EMT is involved in various biological processes, such as embryogenesis and tissue repair, it plays a key cellular role in the development of fibrotic disorders of mature organs, which are often the outcome of pathological chronic disease. Indeed, in animal models, inhibition of EMT has proven beneficial in attenuating the progression of tissue fibrosis [7,8].

Fibrosis results from excessive fibrous connective tissue deposition; in particular, it is followed by the dysregulated production of extracellular matrix (ECM) proteins such as type I collagen and fibronectin by myofibroblasts [9]. There is also evidence that myofibroblasts, key players in the remodelling and maturation phases of wound healing, are derived from resident epithelial cells that have been transformed through EMT to synthesize ECM components. The accumulation of fibrotic components can cause malfunction and failure of the organ affected [10,11,12]. Nevertheless, fibrosis is the final, common pathological consequence of many chronic inflammatory diseases in which multiple pro-inflammatory signalling can switch on the EMT program, activating a pathological fibrotic state. Thus, the origin of the fibrosis process is often described as an unresolved inflammatory response [1,12]. Several studies in cultured cells in vitro and in animal models, as also in human specimens in vivo, have provided evidence that the EMT process is involved in fibrotic disease of multiple organs, such as kidney, liver, lung, intestine, and salivary glands [13,14,15,16,17,18,19,20,21,22,23]. Given the interesting parallels between the regulation landscape of EMT and critical wound-healing processes, it is quite plausible that prolonged activation of the EMT process, in the context of the response to injury, may induce an inflammatory condition and trigger severe fibrosis that culminates in non-healing wounds of several epithelial tissues, thus provoking the destruction of the organ tissue architecture; conversely, inflammation is a potent inducer of EMT, thus suggesting that the two phenomena may sustain each other [17,18].

Fibrosis is also a main pathological characteristic of various chronic autoimmune diseases. In fact, much research on EMT-dependent fibrosis relating to autoimmune conditions has been carried out. It is not clear whether EMT plays a key role in autoimmune disorders, nor whether epithelial cells can undergo EMT during these pathological conditions [7,24,25]. Recently, authors have demonstrated that, in the joints of patients affected by rheumatoid arthritis (RA), there is an abnormal expression of various factors responsible for the EMT process [26,27,28,29]. In addition, recent findings highlighted EMT as a main feature in intestinal fibrosis linked to inflammatory bowel disease (IBD), a severe and common complication in patients affected by this disorder, that includes ulcerative colitis and Crohn’s disease [13,30]. In parallel with what has been observed in IBD, EMT plays a key role in the renal fibrosis features of systemic lupus erythematosus (SLE) nephritis [21,31,32]. The concept that organ failure triggers the inflammatory wound healing cascade, and that pathologically persistent inflammation is closely associated with severe fibrosis, was recently linked to atrophy and fibrosis of the salivary glands (SGs) [17,33,34,35]. It occurs after several episodes of inflammatory states following chronic infections in the glands, as in primary Sjögren’s syndrome (pSS) a progressive systemic autoimmune disease [17,25,35].

In view of the large volume of data present in the literature, this review is focused on the latest news about the relevance of the transforming growth factor (TGF)-β signalling pathway in the regulation of epithelial cell plasticity during the pathogenesis and progression of chronic inflammatory diseases characterized by intense pathological fibrosis, as well as on the ability of TGF-β to promote EMT-induced fibrosis in several autoimmune conditions.

## 2. TGF-β Activation and Signalling

The TGF-β signalling pathway is known to act physiologically as a regulator of embryogenesis, adaptive and innate immunity, cancer development, inflammation, and fibrosis [36]. However, perturbations in TGF-β signalling determine TGF-β switching, accelerating the development or progression of cancer, congenital defects and fibrosis in chronic inflammatory autoimmune conditions [35,37]. During the course of chronic autoimmune diseases, in fact, the activation of fibrogenic mediators produced by inflammatory and epithelial cells was observed. In this context, TGF-β1 emerged as a crucial factor and fibrogenesis, in the majority of the organ where it occurs, is characterized by persistent inflammation, altered interactions between epithelial and mesenchymal cells, and fibroblasts proliferation [38,39].

One of the hallmarks of excessive pathological fibrogenesis is the acquisition by fibroblasts of a highly activated myofibroblasts phenotype characterized by contractile cells that express α-SMA, exhibiting dysregulation of the ECM composition and structure. The recruitment of additional immune cells into the fibrotic tissue amplifies the fibrotic response, because these recruited cells can also secrete a variety of chemokines, cytokines, and growth factors responsible for the differentiation of other myofibroblasts and stimulation of ECM deposition [40,41]. Great attention is paid in the recent literature to the immunological functions of TGF-β; indeed, it exerts a broad anti-inflammatory activity and is considered to be an immunosuppressive agent [42,43]. Complete gene silencing of TGF-β1 in mice could lead to death owing to a multi-organ inflammatory syndrome [44,45]. However, not all TGF-β effects are suppressive; indeed, TGF-β has the capacity to induce either immunosuppressive or inflammatory events, depending on the molecular situation. For example, the Th-17 effector cells are able to acquire their pathogenic function through the mediation of TGF-β and IL-6 [46] and this has been linked to hyperactivation of the immune system caused by the release of inflammatory factors that could ultimately lead to autoimmunity conditions.

Traditionally, TGF-β is a multifunctional cytokine produced by immune cells. TGF-β1 is the prevalent isoform normally found in plasma [47] and ubiquitously bound to ECM proteins in mammalian tissues [47]. Interestingly, large amounts of TGF-β1 are produced by platelets and bones [48,49]. Unlike other cytokines, TGF-β1 is secreted in a latent form that consists of its dimeric pro-peptide [known as the latency-associated peptide (LAP)], and a latent TGF-β-binding protein (LTBP). This tripartite complex of TGF-β, LAP, and LTBP is called the large latent complex (LLC) [50]. TGF-β activation requires proteolysis by plasmin (a factor of the fibrinolytic system) and other proteases. At least in some cases, plasmin-mediated activation occurs on the cell membrane. For instance, the mannose-6-phosphate/insulin-like growth factor II receptor (M6P/IGFII-R) binds LAP-TGF-β on the surface of monocytes and, acting with the urokinase receptor and plasminogen to generate plasmin, determines the activation of the latent TGF-β [51] However, as reported in the literature, other molecules such as platelets expressing thrombospondin 1 (TSP-1), and αvβ6 integrin, an epithelial-cell membrane protein, can also bind and activate LAP-TGF-β [52,53]. Interestingly, the matrix metalloproteinases 2 and 9 (MMP-2 and MMP-9) have been implicated as activators of TGF-β following the involvement of the CD44 hyaluronan receptor [54]. This mechanism, based on the interactions of CD44, MMPs, and TGF-β on the cell membrane, seems to affect cancer cell motility, invasion, and metastasis [55].

## 3. TGF-β Mediates EMT-Dependent Fibrosis

The induction of EMT by TGF-β was first recognized in cell culture. Upon TGF-β treatment, epithelial cells changed from a cuboidal to an elongated spindle shape, and showed a decreased expression of epithelial markers and enhanced expression of the mesenchymal markers fibronectin and vimentin [56]. These changes were accompanied by an increased motility. Consistent with their binding to the same receptor complexes, TGF-β1, TGF-β2, and TGF-β3 share the capacity to induce EMT in epithelial cells [57,58]. Subsequent studies have demonstrated an involvement of TGF-β and TGF-β-related proteins in EMT during normal development and in pathological processes. Fibrosis is characterized by an excessive accumulation of extracellular matrix in the affected tissue; the fibrotic process occurs in various organs and although it can have multiple causes, organ fibrosis typically results from chronic persistent inflammation induced by numerous different stimuli [59]. TGF-β1 is a most important factor involved in the activation of tissue fibrosis, as demonstrated in liver [60], in kidney [32], and in lung [61]. TGF-β1 may generate signals that trigger the EMT program in epithelial cells whose morphology is transformed to that of mesenchymal cells. They produce extracellular matrix components, enriched with fibroblasts in the fibrotic wound, and thus participate in EMT-dependent fibrotic tissue formation [37,62]. When excessive fibrotic production occurs, owing to its profibrotic effects, TGF-β contributes to an excessive tissue fibrosis, leading to organ dysfunction and failure [63]. Indeed, inflammation is recognized as the earliest stage of fibrosis, and emerging data point to the potential role of chronic long-term inflammation as responsible for the development of fibrogenesis [64,65].

During development and in the context of different morphogenetic and/or pathological events such as carcinogenesis, and fibrosis, TGF-β exerts a primary role, inducing various effects depending on the specific cellular context and specific isoforms involved [66]. The first evidence of a role of TGF-β1 in EMT was derived from studies conducted in normal mammary epithelial cells [67]. Since these preliminary studies, TGF-β1 has been shown to mediate EMT in numerous different epithelial cells derived from the lung, liver, lens, or kidney [68,69]. TGF-β is considered to be the prototypical cytokine for the induction of EMT, whereas the effects of other molecules reveal a cell context-dependent mechanism of action [69]. Major evidence of the action of TGF-β on triggering EMT also derives from studies conducted in vivo, especially by means of modulating the TGF-β-dependent Smad pathway in animal models. Knockout of the Smad3 gene or the employment of Smad7, which works as an antagonist of TGF-β signalling, or of bone morphogenetic protein-7 (BMP-7) acting in a Smad-dependent manner, offsets EMT in mice [70,71]. It was also shown to slow and reverse uncontrolled fibrosis processes in renal and lens epithelia [18,72]. During pulmonary fibrosis, potential sources of lung fibroblasts include the proliferation of resident lung interstitial fibroblasts, progenitor cell differentiation, as well as the transition of epithelial cells to a fibroblast phenotype through the EMT process [4]. Confirming this hypothesis, several authors clearly demonstrated that, in vivo, approximately one third of the lung fibroblasts able to release the fibrotic protein S100A4 derive from the lung epithelium after the administration of bleomycin, widely used as an inducer of pulmonary fibrosis in animal models [73,74].

The direct involvement of TGF-β in the EMT-dependent differentiation of lung fibroblasts, in vivo, was recently demonstrated by the detection of increased active TGF-β1 in transgenic mouse permanently β-galactosidase stained alveolar epithelial cells. In this model, X-gal-positive cells underwent phenotypical changes of EMT, expressing the myofibroblast marker α-SMA, while most of the cells identified as X-gal-positive fibroblasts expressed vimentin, indicating an epithelial origin [75].

## 4. TGF-β Activates Smad and Non-Smad Signalling Pathways

**Figure 2:**
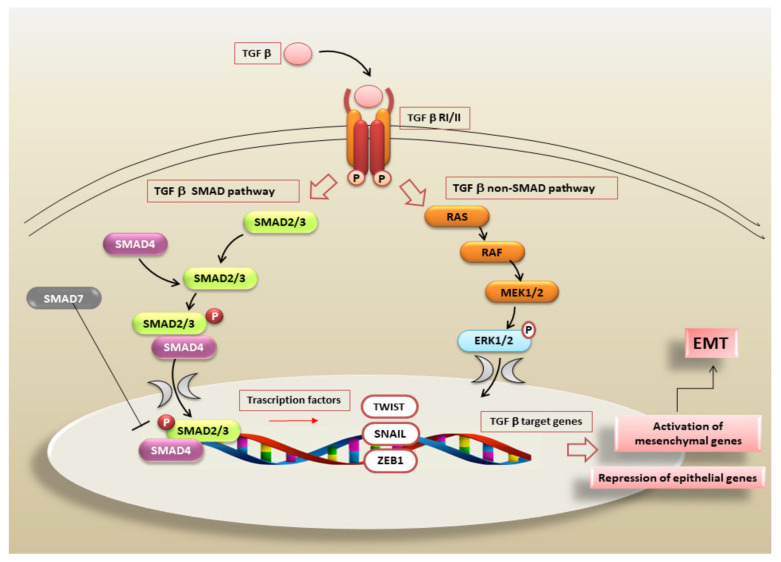
TGF-β, a key mediator in the pathogenesis of fibrosis. TGF-β, through the downstream factors, exerts biological activities on different cell types, acting as a central molecule in the activation of the fibrotic program.

The TGF-β1/SMAD/Snail pathway is a particularly interesting system activated during the TGF-β-stimulated EMT that leads to the EMT-dependent fibrotic process in a number of diseases [25,60,61,76]. All three forms of TGF-β use the same receptors: type I (RI, or ALK5), type II (RII), and type III (RIII, or betaglycan) [77]. After TGF-β binding to RIII, TGF-β is recruited to RII and RI is phosphorylated, forming a heterotetrameric serine/threonine kinase complex [78]. The main signalling pathway induced by TGF-β is primarily mediated by the phosphorylation of regulatory SMAD2/3 proteins to form the receptor-mediated-Smad (R-Smad)/co-regulatory Smad4 complex [79]. This SMAD complex accumulates in the nucleus where, through the activation of transcription factors, it stimulates the transcription of target genes [80]. The transcription of the genes encoding the zinc finger transcription factor Snail, for example, was successfully induced [81]. Snail plays an active role in guiding the EMT process which involves the loss of E-cadherin and claudins (epithelial markers) and concomitant upregulation of vimentin and fibronectin (mesenchymal markers) (Figure 2) [35,82]. As clearly demonstrated by Peinado et al., Snail works as a strong repressor of the expression of the epithelial marker E-cadherin, in an experimental model consisting of Madin–Darby canine kidney cells treated with TGF-β1. In these cells, a TGF-β1-dependent EMT program was activated, suggesting that Snail is a direct target of TGF-β1 signalling [83].

Considerable evidence underlines the importance of SMADs in TGF-β-EMT-dependent fibrosis: Smad3 gene knockdown blocks EMT activation in response to TGF-β or mechanical stress in renal tubular epithelial cells [84], and determines a reduced migratory capacity of keratinocytes in response to TGF-β [85]. Compared with Smad3, Smad2 may play an antagonistic role in the EMT process in vivo. In human skin cancer, Smad2 loss was associated with dedifferentiation, and the loss of E-cadherin. Moreover, underexpression of Smad2 is frequently detected in human skin cancer patients. Among EMT-associated genes, Snail overexpression appears to be correlated with Smad2 loss-induced EMT [86]. This has been explained by suggesting that Smad2 could either compete with or impede the ability of Smad4 to bind Smad3 and activate the Snail promoter. Therefore, Smad2 loss confers a greater binding capacity of the Smad3/4 complex to the promoter of the Snail gene, thus enhancing the progression of EMT [86]. Supporting this explanation, Ju et al. reported that Smad2–/– hepatocytes derived from knockout mice underwent the EMT activation program and mesenchymal cells appeared, while Smad3–/– hepatocytes retained their epithelial characteristics and did not exhibit EMT-associated molecular alterations [87]. Like that of Smad3, the role of Smad4 resulted indispensable for EMT activation. Smad4 expression knockdown, performed by means of RNA interference, or transfection with a dominant negative mutant form of Smad4, resulted in preserved E-cadherin expression in a pancreatic cancer cell line [88], suppression of type I collagen synthesis in vitro, fibrotic process features, and decreased bone metastasis in vivo [89]. The same observations were made in a study conducted on adenocarcinoma, demonstrating that genetic ablation of Smad4 leads to the preservation of epithelial markers and inhibition of EMT [90].

Recently, inhibitory Smads (I-Smads) were identified, whose role was to inhibit TGF-β family signalling through multiple mechanisms, based on the interactions with activated type I receptors and activated R-Smads. Smad6 and Smad7 are classified as I-Smads, since they function as negative regulators of the TGF-β-induced EMT [91,92,93]. I-Smads are able to interfere with the interactions between R-Smads and type I receptors, to cause down-regulation of cell type I receptors expression, to prevent the complex formation by R-Smads and co-regulatory Smads, and to control Smads-dependent transcriptional regulation in the nucleus [94]. Smad7, in particular, blocks TGF-β-induced EMT in multiple tissues [95,96]. Nevertheless, Smad7 is closely linked to the activation of TGF-β during the course of chronic inflammatory diseases, which often trigger EMT-dependent fibrosis. It was recently demonstrated that Smad7 is overexpressed in the colonic mucosa when chronically inflamed, and in precancerous conditions. Confirming this evidence, inhibiting Smad7 through the oral administration of Smad7 antisense oligonucleotide in vivo decreased inflammation in mice with colitis induced by haptenating reagents [97]. Knockdown of Smad7, in fact, restores TGF-β1 activity, thus suppressing inflammatory cytokines release and leading to the resolution of colitis in mice [97]. Consistently, oral administration of anti-Smad7 antisense oligonucleotide to patients with active Crohn’s disease induces clinical remission [98].

In addition to Smads pathways, TGF-β1 also utilizes a multitude of intracellular non-canonical, non-Smads TGF-β signals that are generally triggered by the binding of ligands not belonging to the TGF-β family to tyrosine kinase receptors [99]. The activated TGF-β1 receptors induce a response through signal transducers such as the mitogen-activated protein kinase (MAPK) pathways, for example, including extracellular signal-regulated kinases (Erks), c-Jun amino terminal kinase (JNK), p38 MAPK, as well as IκB kinase (IKK), phosphatidylinositol-3 kinase (PI3K) and Akt, and the Rho family GTPases, that could finally converge on the Smad activating cascade [76,99]. The involvement of these non-Smad pathways in TGF-β1/EMT-dependent fibrosis was recently demonstrated by the use of chemical inhibitors directed against one or several of these pathways. The PI3K/Akt pathway is implicated, besides EMT, in TGF-β-mediated fibroblast proliferation and morphological EMT-dependent transformation. The use of a chemical inhibitor, such as imatinib mesylate, prevented TGF-β1/EMT-dependent fibrosis in the lung [100]. In addition, using specific small-molecule inhibitors blocking the MAP kinase or PI3 kinase pathways, Janda et al. identified the Raf-MAP kinase pathway as synergistically involved in TGF-β signalling, accelerating tumorigenesis and metastasis in EpH4 mammary epithelial cells through the induction of EMT [101]. Based on the scientific evidence, regulation of the immune function and inhibition of EMT may be among the most important mechanisms for the prevention of cancer and chronic inflammatory diseases progression. Identifying TGF-β antagonists blocking both Smad- and non-Smad-dependent pathways, which are clearly only partially distinct molecular mechanisms, is a priority research goal.

## 5. TGF-β Induces Epithelial Cell Plasticity

The epithelium includes various highly specialized cells that play critical roles in almost all biological processes [102,103]. Indeed, epithelial cells constitute a protective barrier that serves to cover the external and internal surfaces of the body cavities. The role of the epithelium is equally important in supplying the first line of protection against the external environment, against infections, in particular, while simultaneously allowing the exchange of substances that are essential to preserve tissue homeostasis [68]. Recent evidence has linked the development of tissue fibrosis to an inappropriate reactivation of the EMT program [104,105]. In mature tissues, EMT is also switched on in damaged epithelia to facilitate healing, remodelling, and repair in response to tissue injury [68]. Thus, completely differentiated epithelial cells exert a silent transcriptional EMT process that can be restarted in response to multiple specific extracellular signals, one of them being the multifunctional cytokine, TGF-β [104,106,107]. Interestingly, these same cellular and morphological characteristics are observed in cells subjected to a pathological EMT program, which determines the development of various human diseases, such as chronic inflammation, rheumatoid arthritis, and chronic fibrotic degenerative disorders of the lung, liver, and kidney [61,103,104,106,107,108]. Recent studies indicate that numerous pathological actions of TGF-β could be referred to its ability to modulate epithelial cell plasticity, inducing alterations in the phenotype of several cell populations [109]. Cell plasticity includes the interconversion of epithelial cells within a tissue [61] and the reversible capacity to change their phenotype and to acquire features of other cell types [109,110]. The epithelial cells are reported to trans-differentiate into myofibroblasts through the EMT program activated by chronic inflammation in different fibrosis models [4]. After TGF-β stimulation, the cells present genetic and epigenetic changes, as well as a variety of tissue remodelling events such as that of the cytoskeleton, which modify their phenotype and functions. Importantly, EMT induction by specific stimuli like TGF-β was originally demonstrated by Miettinen et al. [67], who noted that normal mammary epithelial cells acquire fibroblastic phenotypes in response to TGF-β. Further advances suggested that the accumulation of myofibroblasts, a prevalent ECM production source, is a critical step in the progression of renal fibrosis [104]. However, the origin of myofibroblasts is still controversial; it has been suggested that they may be derived from epithelial cells. Additionally, it is generally accepted that local fibroblasts can differentiate into myofibroblasts after TGF-β stimulation [111,112]. TGF-β administration induces the differentiation of epithelial cells and endothelial cells into myofibroblast-like cells, whereas blocking TGF-β/Smad signalling with inhibitors reverses EMT or EndoMT process [18,21,113,114]. More light on the role of epithelial cells during TGF-β-dependent pulmonary fibrosis has been shed in recent decades [115,116,117,118,119]. In the lung, TGF-β is expressed by multiple cell types, including epithelial cells, whose levels resulted elevated in animal models and in patients affected by idiopathic pulmonary fibrosis (IPF) suffering from widespread, progressive fibrotic lung disease [115,120]. IPF is provoked by progressive, irreversible scarring of the lung, injury to the alveolar epithelium, the recruitment of fibroblasts and trans-differentiation into myofibroblasts, and subsequent accumulation of the ECM in response to the resulting aberrant activation of repair mechanisms [115,116]. Pulmonary expression of TGF-β is sufficient to trigger progressive fibrosis in animal models such as rodents [121], while blocking TGF-β signalling inhibits pathological fibrosis [122]. It was also shown in vitro that TGF-β promotes the mesenchymal transition of epithelial cells, and that myofibroblasts isolated from patients affected by IPF show permanent fibroblastic features and also secrete their own TGF-β. This triggers further ECM components through Smad-dependent signalling, which perpetuates the altered wound-healing process [123,124,125,126].

Interesting findings have highlighted that TGF-β plays crucial roles in the development of liver fibrosis, modulating several phases of the disease and regulating the plasticity of the epithelial cell pool, including hepatocytes [60]. Hepatocyte plasticity could play a relevant functional role during the progression of chronic liver diseases. Moreover, after TGF-β stimulation, the primary adult hepatocytes lose their junctions and apical–basal polarity, repress epithelial marker genes, activate mesenchymal factors, and thus transdifferentiate into cells with a fibroblastic-like morphology [127,128]. Indeed, hepatocyte-derived fibroblasts are an additional, significant lineage of mesenchymal cells that contribute to the progression of liver fibrosis. Importantly, Zeisberg and collaborators showed, in vivo, that adult hepatocytes can undergo an EMT program after treatment with TGF-β, boosting the fibroblasts population during liver fibrosis [16]. The role of hepatocytes during liver fibrosis subjected to TGF-β stimulation in vivo had been previously investigated in a transgenic animal model which overexpresses SMAD7 (inhibitor of the pathway) specifically in hepatocytes. These transgenic animals had a reduced TGF-β signalling and EMT, showing less ECM accumulation, a less marked fibrotic process, and hence milder liver injury [129].

**Figure 3:**
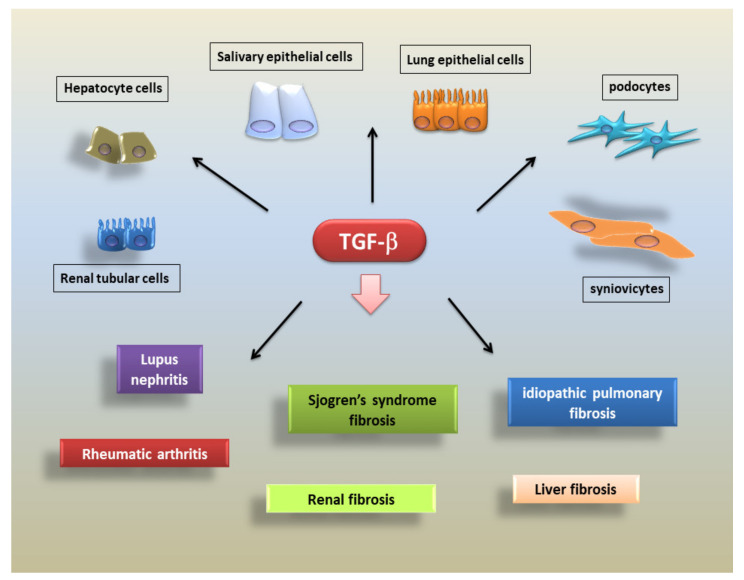
Schematic overview of TGF-β/SMAD and non-SMAD signalling. TGF-β triggers cellular responses by binding and activating two transmembrane Ser/Thr protein kinase receptors, denominated TGF-β type I (TβR-I) and type II (TβR-II). In the canonical SMAD pathway, activation of these ligand–receptor complexes lead to the phosphorylation and activation of SMAD2/3 which, in turn, binds to cofactor SMAD4. The trimeric SMAD complex translocates into the nucleus and cooperates with other transcription molecules to regulate target gene expression. The activation of transcription factors such as Snail, Twist, or ZEB promotes the prolonged induction of EMT, repressing epithelial marker genes and activating genes linked to the mesenchymal phenotype. Feedback regulation is mediated by SMAD7 which interferes with the binding of SMAD2/3 to TGF-β receptors. In the non-SMAD pathway, TGF-β receptors activate other pathways that contribute to induce the EMT program. The RAS–RAF–MEK–ERK MAPK signalling cascade is a major non-canonical pathway activated by the TGF-β/ TGF-βRI/II complex. Once activated, ERK1 and ERK2 MAPK can facilitate EMT by increasing the expression of EMT transcription factors.

Important emerging evidence from studies of renal fibrosis suggests that more than a third of all disease-related fibroblasts originate from tubular epithelia at the site of damage. Indeed, TGF-β can promote renal fibrosis through a cellular interaction mechanism and, interestingly, the injured epithelium releases TGF-β, which is able to activate trans-differentiation into pericyte-myofibroblasts [130]. The renal tubular epithelium is often the cellular target of injury, and there is also increasing evidence supporting its role as a relevant, key promoter of tubule–interstitial fibrosis. Elevated expression of TGF-β and its receptor has been demonstrated in the altered epithelium in various models of glomerular and renal tubular damage [131]. In this context of damaged renal tubules, epithelial TGF-β activated signalling induces pathological fibrosis affecting renal tissue integrity and determining abundant synthesis of the ECM proteins by both tubular epithelial cells and fibroblasts [132]. TGF-β triggers the differentiation of damaged renal epithelial cells into mesenchymal fibroblastic cells. This model of pathogenic events mediated by TGF-β includes the disruption and disassembly of desmosomes and E-cadherin in the renal epithelial cells and induces the expression of mesenchymal-like proteins such as α-SMA and vimentin. Remodelling of the actin cytoskeleton occurs, and cell motility increases [133]. Intriguingly, the current scientific focus on fibrogenesis offers the first demonstration that the level of TGF-β1 is increased in SGs tissues derived from biopsies of patients affected by pSS, an autoimmune disease characterized by a severe inflammatory condition that contributes to an excess of fibrotic salivary tissue. This compromises the functional morphology of SGs, owing to evident morphological changes of the glandular epithelium (Figure 3) [17,35].

## 6. TGF-β-Induced EMT: Molecular Mechanisms in Rheumatoid Arthritis

Rheumatoid arthritis (RA) is an autoimmune and chronic inflammatory disease featuring synovial hyperplasia and progressive joint destruction [134]. Synovial hyperplasia is caused by the influx and proliferation of inflammatory cells, due to an increased neo-angiogenesis within the hyperplastic tissue [135], as well as by the increased proliferation and survival of resident cells [135].

In this context of an enhanced, prolonged production of inflammatory cytokines, TGF-β and NF-κB signalling are crucial regulatory pathways in RA [136]. In RA, TGF-β1 performs actions that range from a pro-angiogenic role in the synovial membrane (SM) [137] to the induction and synthesis of pro-inflammatory cytokines [138], MMPs [139], and various agents responsible for fibrinolysis and tissue remodelling, such as aggrecanase [140] and urokinase-type plasminogen activator [141]. The importance of TGF-β in the pathogenesis of RA has been highlighted by numerous studies conducted on animal models. The abundant expression of TGF-β1, 2, and 3 and of TGFBR1 and -2 in rat synovium, acts as a potent inducer of collagen-linked arthritis (CIA) [142], while the intra-articular injection of TGF-β1 or TGF-β2 induces symptoms typical of RA, resulting in synovial inflammation and hyperplasia [143]. On the contrary, when TGF-β activity was blocked, the neutralization of acute and chronic RA symptoms triggered by streptococcal cell walls (SCW) occurred [144]. Confirming these observations, an adenovirus-mediated overexpression of TGF-β1 in rabbit knees led to fibrosis and muscle oedema mediated by an increased glycosaminoglycan release and nitric oxide production [145].

As clearly described in the paragraph describing TGF-β-mediated molecular pathways, TGF-β, through both type I and type II receptors, regulates TGF-β-responsive genes transcription by inducing the phosphorylation of Smad2 and Smad3 [76,80,146]. In addition, TGF-β induces Smad7 expression, which negatively regulates TGF-β/Smad signalling via a feedback inhibitory mechanism [92]. Based on these assumptions, in RA patients, the loss of inhibitory Smad7 was associated with a marked activation of TGFβ/Smad3 signalling and the development of arthritis [146]. Furthermore, in RA, not only did the inhibition of Smad7 promote TGFβ/Smad3 signalling and tissue fibrosis, but it also markedly enhanced NF-κB-driven inflammation [146]. Supporting the protective and inhibitory role of Smad7 in tissue fibrosis and inflammation, the Smad7 deficiency observed in RA patients enhanced NF-κB signalling, Th1/Th17 differentiation, and synovial inflammation, probably through the hyperactivation of the TGF-β/Smad3-IL-6 molecular pathway, which is no longer controlled because of Smad7 inhibition [146]. It is not yet clear whether it is Smad7 that has an effect on NF-κB activity or else it is NF-κB that acts by regulating the expression of Smad7. Interestingly, it was demonstrated that NF-κB is required for the transcriptional activation of Smad7 by pro-inflammatory cytokines release. Smad7, whose expression is consequently increased, repressed the TGF-β/SMAD pathway through its direct link with TGF-β ligand-receptor I binding, since the TGF-β receptor complexes resulted progressively occupied due to their interaction with Smad7 [147].

In contrast to the above-mentioned studies, several authors reported beneficial effects of TGF-β in RA. The systemic administration of TGF-β1 ameliorates SCW-induced RA, in terms of cellular infiltration and joint erosion [148]. In addition, evaluating the cytokine expression during CIA, a strong upregulation of TGF-β1/2 in the disease remission state was demonstrated, suggesting that TGF-β could exert an anti-inflammatory regulation of T-cells in RA [149].

The great variety of experimental data collected prompted many authors to investigate a possible link between TGF-β expression in RA and EMT, in view of the key role of TGF-β in EMT induction. During EMT, the phenotype of cells changes, becoming more aggressive, invasive, and resistant to apoptosis [4]. These processes, which are characteristic of metastatic processes in tumours, determine pannus tissue invasion and destruction in RA [27]. In RA patients and in the CIA mice model, the synovial membrane or synovial fluid shows increased levels of EMT-inducing molecules, including TGF-β [150]. Some highly innovative studies reported that TGF-β was up-regulated by transglutaminase 2 (TG2), an important enzyme in the regulation of EMT and ECM composition/stiffness/degradation [151]. TG2 was shown to play a key role in invadosome formation by fibroblast-like synoviocytes (RA-FLS), through its ability to cross-link with the ECM and to determine the activation of TGF-β [152]. This complex network of inducers/pathways leads to the activation of key EMT regulators, such as the transcription factors Snail/Slug, Twist, Zeb, and E47, which organize a concerted modulation of EMT [26]. Multiple components that regulate fibroblast formation are present in the RA synovium, either as genes or proteins; for example, large amounts of both latent and activated TGF-βI and II are found in RA synovial membranes and synovial fluids [29,153]. Mesenchymal cells that stain positive for an anti-phosphorylated Smad 2/3 antibody are evident in RA articular tissues [154]. In addition, myofibroblasts or other cell types that react with an antibody to αSMA are detected in a proportion of synovial fibroblasts, but absent in normal or even osteoarthritis synovium [155]. Common MMPs can further stimulate fibroblast formation through EMT. For example, in healthy epithelial cells MMP3 induces a fibroblast-like phenotype through the transcriptional upregulation of Rac-1b, as well as an increased production of reactive oxygen species. These are responsible for an enhanced expression of the Snail transcription protein in the synovium and cell lines derived from RA patients [156]. These recently published findings support the hypothesis that Snail mediates ECM degradation by human and rat synovial cells, and is involved in cartilage degradation detected in a CIA model [152]. However, despite growing evidence of an involvement of TGF-β/EMT in RA, some important reservations need to be kept in mind, because in the synovium few cells have epithelial characteristics, and classical E-cadherins are poorly expressed due to the lack of a basement membrane [157]. These key points need to be solved to clearly identify the critical role likely played by EMT in RA joints. Nevertheless, the presence of α-SMA in the synovial lining layer of RA patients and the expression of fibrotic factors in healthy FLSs after stimulation with synovial fluid from RA patients [158] indicate that a modulated process like EMT might play a role in the development of RA synovium.

## 7. Involvement of TGF-β Signalling and EMT in Autoimmune Diabetes

Autoimmune diabetes determines severe long-term effects on various organs, such as the eyes, kidney, heart, brain, liver, and lung [159]. At the tissue level, diabetes has been found to induce several pathological changes, including a chronic inflammatory state and fibrosis [160]. Indeed, multiple findings have demonstrated that patients suffering from diabetes, types both 1 and 2, present complications that involve the lung through the induction and progression of fibrotic infiltrations into the pulmonary tissue [159,161,162,163]. Inflammatory cell infiltrations and elevated levels of ECM proteins in the lung induce an inflammatory process that leads, as a consequence, to the development of pulmonary fibrosis and tissue injury. EMT is an important cellular program that leads to the development of pathological fibrosis in the lung, and diabetes can induce EMT through the persistent effects of hyperglycemia [164]. In addition, EMT triggered by diabetes is modulated mainly by the upregulation of TGF-β1 and Snail, an EMT transcriptional factor, leading to an elevated accumulation of ECM proteins in the pulmonary tissue and the downregulation of epithelial markers such as ZO-1 and cadherin [165,166,167,168]. In a recent study, to elucidate the pathological effects of TGF-β1 on the lung, a diabetic animal model that employs multiple administrations of low-dose streptozotocin (STZ) was used to produce diabetes in mice [159]. The activation of TGF-β1 signalling triggered the EMT process in alveolar epithelial cells harvested from control and diabetic mice, which later exhibited a fibroblast phenotype and elevated expression levels of fibrotic mesenchymal markers [6]. These studies offer clear evidence that diabetes triggers inflammation and fibrotic conditions in the lung, modulated by the induction of the TGF-β1 signalling pathway. Upregulation of the TGF-β1 receptor and SMAD2/3 levels in the diabetic cells, as well as increased levels of p38 and ERK, suggested that diabetes-induced fibrosis was mediated by the activation of both SMAD-dependent and SMAD-independent pathways [159].

Furthermore, TGF-β1 signalling pathways are negatively regulated by SMAD7 activation [169], and decreased levels of SMAD7 in the diabetic cells compared to those in the controls led to a delayed response of the lung to the diabetes, and also confirmed the activation of the TGF-β1 signalling pathway in the lung [159].

In recent years, much attention has been paid to the role of TGF-β and its signal transduction in diabetic kidney disease [170,171,172,173,174,175,176], characterized by thickening of the glomerular basement membrane, mesangial expansion, and severe interstitial fibrosis [170]. Several studies have demonstrated an increased expression of TGF-β in cultured renal cells and animal models of diabetic nephropathy [173,175,177]. The TGF-β expression level is increased in the kidneys of diabetic mice during both early and late stages of the disease [176,177]. In STZ-diabetic mice an elevated TGF-β1 expression was observed in the renal cortex and glomeruli, as well as the upregulation of the TGF-β1 receptor gene and protein [176,178,179]. Other studies evaluating the functional role of renal TGF-β signalling yielded interesting findings demonstrating that treatment in vivo of STZ-induced diabetic mice with neutralizing monoclonal antibodies against all three TGF-β isoforms reduced the expression of mesenchymal markers such as collagen and fibronectin, ameliorating renal fibrosis [180,181,182]. Additionally, more focus has been devoted to downstream targets of the TGF-β signalling pathway, showing that this particular effect of TGF-β1 appears to be mediated by Smad3 [183]. This highlights the concept that the latter is a central mediator in TGF-β1 signalling [184,185]. The use of either a Smad3-specific inhibitor or Smad3 gene knockdown consistently confirmed a delayed transdifferentiation of proximal tubular cells and reduced renal fibrosis in diabetic mice [173,186]. These findings suggest that TGF-β1/Smad3 signalling has a critical role in renal fibrosis. In addition, treatment with exogenous TGF-β1 leads to an over-expression of Smad3, which synergistically increases fibronectin and collagen expression, augmenting the efficiency of TGF-β signalling [183]. Findings made in diabetic patients with several stages of nephropathy also underline the importance of renal TGF-β signalling in the disease development [187,188]. All three TGF-β isoforms are increased in both the glomerular and the interstitial spaces of patients with diabetic nephropathy [187,188]. Furthermore, glomerular TGF-β1 mRNA is intensively elevated in biopsy tissues derived from patients with proven diabetic kidney disease. These studies suggest that elevated renal TGF-β expression levels are closely correlated with severe interstitial fibrosis and renal failure.

## 8. TGF-β-Induced EMT in SLE-Dependent Fibrotic Diseases

Although recent literature has paid great attention to the role of TGF-β in Systemic lupus erythematosus (SLE), a clear picture of the role of this cytokine in this disease is still lacking. SLE is a chronic autoimmune disorder characterized by autoantibody production affecting multiple organs [189]. In this autoimmune disease, T cells infiltrate target organs, leading to the accumulation of immune complexes containing autoantibodies. This phenomenon causes an inflammatory response which can be countered by anti-inflammatory cytokines release by macrophages, such as TGF-β1 [190]. Nevertheless, the enhanced TGF-β1 production in tissues induces the activation of local fibrogenesis, ultimately causing severe organ failure [191]. Thus, indirect approaches to manipulate the cytokine network to treat SLE, also involving the control of TGF-β1 levels, need to be made with caution and taking into account a range of possible consequences on the cytokine network regulation mechanism as a whole [187]. Confirmation of the involvement of TGF-β1 in the pathogenesis of SLE was gained with the experimental use of TGF-β1-deficient mice that developed a SLE-like disorder characterized by various autoantibodies production [192]. On the contrary, in a murine model of SLE cells transfection with a vector that encodes TGF-β1, enhanced mice survival and a beneficial effect of TGF-β1 on the disease progression was observed, lessening the disease severity [193]. This observation suggests that TGF-β1 can play a protective role in SLE disease [193], which could explain why, in general, SLE patients produce lower levels of TGF-β as compared with healthy individuals [194,195,196]. Furthermore, TGF-β levels are inversely related to the disease severity, suggesting that this may be affected by TGF-β production [195]. A selection of the latest research on this topic imputes the cause to an impaired production of TGF-β1 by lymphocytes isolated from SLE patients [197]. However, an interesting study made by Xing and colleagues [198] revealed that, in SLE patients, serum TGF-β1 concentrations decrease in parallel with the reduction of peripheral regulatory T cells. On the other hand, other researchers, although they demonstrated a defective TGF-β1 gene transcription in T and B lymphocytes derived from SLE patients peripheral blood, nevertheless detected slightly increased serum levels of TGF-β1 in SLE patients as compared to controls [199]. These apparently paradoxical findings were explained by hypothesizing TGF-β1 production by monocytes and other non-lymphoid cells [199].

Now, is there any current evidence of the activation of a TGF-β-dependent EMT program in SLE that could possibly explain the onset of a fibrotic process in target organs? Kidney disease is one of the most serious manifestations of SLE, and nephritis in SLE is one of the most important causes of morbidity and mortality. TGF-β seems to play a prominent role in SLE-associated renal disease [200], and significantly lower levels of serum TGF-β1 were detected in SLE patients with renal damage. This was supported by subsequent data reported by Jin et al. [201], who detected significantly lower levels of serum TGF-β1 in patients with severe kidney damage. However, as reported above, in SLE patients the TGF-β1 system could presumably undergo activation in the target organ characterized by a local inflammatory condition, even if systemic TGF-β1 levels were decreased. This confirms data collected by Saxena et al. [202], who conducted studies in vitro on a murine SLE model, showing that TGF-β1 concentrations in kidneys and urine were correlated with the degree of chronic kidney disease [202]. Starting from this experimental evidence, several researchers aimed to evaluate the evolution of the kidney disease, by investigating whether renal damage was associated with fibrotic degeneration mediated by TGF-β.

In human glomerular diseases, such as lupus nephritis, TGF-β has been considered a critical tissue injury factor contributing to glomerular lesions associated to focal glomerulosclerosis [203,204]. In these diseases, which are characterized by an excessive synthesis and accumulation of ECM, a significantly increased expression of all TGF-β isoforms and relative TGF-β receptors in the glomeruli, tubule and tubular interstitium has been demonstrated [205]. Interestingly, an evident EMT-dependent fibrosis has been shown in tubular epithelial cells derived from human renal biopsies collected from various cases of renal diseases, including lupus nephritis [206]. As a consequence, TGF-β1 is now widely recognized as a master inducer of pathological fibrosis linked to EMT in renal structures during chronic kidney disease [206,207]. TGF-β1, in fact, downregulates the expression of epithelial factors E-cadherin and ZO-1, through the proteolytic shedding of E-cadherin by MMPs in tubular epithelial cells. In addition, in response to TGF-β1, cultured proximal tubular epithelial cells undergo marked morphological changes accompanied by the de novo synthesis of the mesenchymal marker α-SMA. Furthermore, TGF-β1 induces an increased expression of its downstream mediators MMP2 and MMP9, which alter the tubular basement membranes integrity, allowing the cells undergoing EMT to become migratory and facilitating the invasion of the interstitium [208,209].

## 9. TGF-β-Induced Brain Endothelial Cell Dysfunction in Multiple Sclerosis

Multiple sclerosis (MS) is a chronic neuro-inflammatory disorder which is characterized by an alteration of the blood–brain barrier (BBB) [210]. MS presents an autoimmune component in which numerous blood-derived lymphocytes recognize and attack myelin antigens entering the brain by crossing the damaged BBB [211,212,213]. BBB has a key role in preserving brain homeostasis through the specialized function of brain endothelial cells (BECs). Inflammation of the BECs and loss of their neuroprotective functions is an important feature of this neurological disorder [214,215].

As it seems likely that modifications in BECs junctions determine an increased permeability of the BBB, as seen in MS, some authors have studied the alterations in junction molecules that regulate BECs integrity [216,217]. These are especially important since endothelial junctions have a crucial function in creating a strict barrier preventing the entrance of neurotoxic agents into the CNS [218]. One recent postmortem study examined the distributions of tight junction proteins such as occludin and ZO-1, demonstrating an altered localization of ZO-1 and occludin in blood vessels of active MS lesions [219]. Multiple signalling pathways are critical for the integrity of the endothelium, and loss of these crucial signalling systems can result in a process named the endothelial to mesenchymal transition (EndoMT) in which endothelial cells present degradation of the basement membrane and loss of cell-cell contact, acquiring a migratory phenotype [220,221,222,223]. Under these conditions, the EndoMT may have a crucial role in BBB impairment in MS [224]. Interestingly, a presumptive association between the EndoMT and inflammation-induced BBB dysfunction was demonstrated [225]. Notably, TGF-β1 was localized in chronic active MS lesions, thus underlining its regulatory role during MS disease progression [226]. A recent work demonstrated a key role of SNAIL, as a repressor of tight junction gene expression in BECs [225]. Increased SNAIL expression was shown to mediate BECs impairment [227]. Troletti and colleagues hypothesized that TGF-β1-induced BECs dysfunction in MS might be due to BECs trans-differentiation through the EndoMT [225]. The authors also demonstrated that SNAIL mRNA levels and protein were significantly boosted by TGF-β1 in MS lesions [225]. Furthermore, mRNA levels of multiple junctional factors, such as claudin-1 and claudin-5, resulted significantly reduced. Lastly, mRNA levels of key mesenchymal components like fibronectin and vimentin were significantly increased in MS lesions. Moreover, stimulation of human BECs with TGF-β1 promoted the EndoMT, demonstrating the involvement of TGF-β in BECs during MS [225].

## 10. TGF-β1-Mediated EMT-Dependent Fibrosis in Sjögren’s Syndrome

Recently, EMT has been identified as a new source of cells with a mesenchymal phenotype involved in the progression of fibrotic diseases [3,17,35]. During chronic inflammatory diseases, tissue injury derived from the activation of a cascade of events leads to an excessive accumulation of ECM components. This phenomenon is closely linked to fibrogenesis and was recently associated with atrophy and fibrosis of the SGs [228,229]. Indeed, SGs fibrosis was demonstrated following recurrent episodes of inflammation, causing a decreased secretory function of SGs inducing hyposalivation and xerostomia, as in pSS [33]. pSS is a frequent systemic rheumatic autoimmune disorder (prevalence 0.5–2%), whose cardinal features are lymphocytic infiltration of the salivary and lachrymal glands which leads to dry eyes and mouth, so that it is also known as the “Sicca Syndrome” [230]. Fibrosis has been observed in the SGs of pSS patients [33], ascribed to tissue damage and chronic inflammation [33]. There is currently no effective treatment for pSS fibrosis, which may be attributed in part to lack of a clear underlying mechanism, although the prevailing hypothesis suggests the implication of fibrogenic mediators produced by inflammatory and epithelial cells, and, in particular, TGF-β1 [107]. TGF-β1 was found to promote SGs epithelial cells (SGEC) transition to form mesenchymal cells with a myofibroblast-like phenotype in inflamed SGs, thus contributing to SGs fibrosis [231,232,233]. Experimental in vitro studies demonstrated, in fact, that SGEC collected from healthy volunteers treated with TGF-β1 adopted a more fibroblast-like morphology with a marked weakening of cell-cell adhesion. By contrast, when exogenous TGF-β1 activity was inhibited by the use of TGF-β1-pathway inhibitors, healthy SGEC maintained the morphological and structural features of epithelial cells, displaying the classic cobblestone morphology and growth pattern. Furthermore, pSS SGs biopsy specimens are characterized by an elevated expression of TGF-β1 in the glandular epithelium [234].

**Figure 4:**
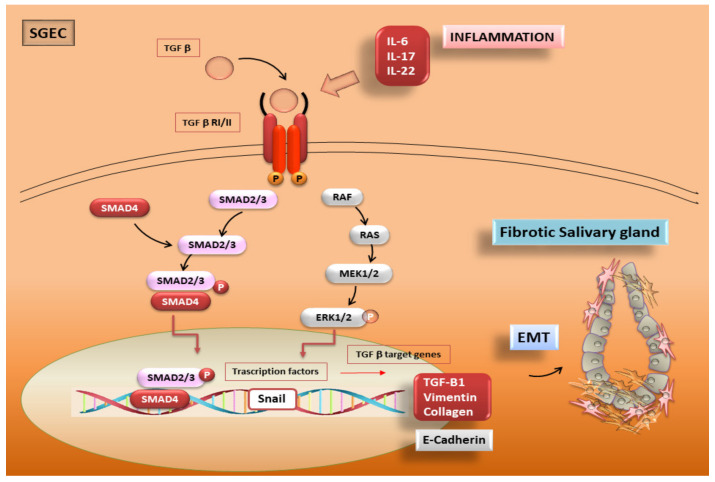
TGF-β/EMT signalling in pSS. TGF-β activates both the canonical SMAD2/3 pathway and the non-canonical MAPK pathway, triggering the EMT process in healthy SGEC. An inflammatory microenvironment including pro-inflammatory cytokines such as IL-17, IL-22, and IL-6 may induce EMT through the TGF-β/SMAD and non-SMAD signalling pathways. TGF-β exerts its cellular effects by linking to the TGF-β type I/ II receptor, and this binding leads to the activation of SMAD2/3 canonical signalling. Phosphorylated SMAD2/3, with cofactor SMAD4, form a heteromeric complex, which translocates to the nucleus to mediate signalling events linked to EMT activation. The activation of the transcription factor Snail and the induction of EMT markers have the consequence of upregulating the mesenchymal markers Vimentin and Collagen Type 1 and downregulating the epithelial marker E-Cadherin. During the activated EMT program, salivary epithelial cells exhibit dramatic morphological changes and the acquisition of mesenchymal properties, including an increased migratory capacity and contractility. Finally, these mesenchymal cells become myofibroblasts, which are responsible for progressive SG fibrosis. Alternatively, TGF-β can deliver signals via non-SMAD pathways, such as the MAPK signalling cascade including phosphorylated ERK1/2. The ERK1/2 signalling pathway has a complex and crucial correlation with the TGF-β1 system in controlling the EMT process.

There is evidence supporting the idea that the aberrant upregulation of TGF-β1 in the pSS SGs causes EMT via the activation of the TGF-β1/SMAD/Snail signalling pathway. Sisto and colleagues demonstrated that TGF-β1, pSMAD2/3, and SMAD4 proteins are widely expressed in the pSS tissue in patients, at higher levels than in healthy SG tissues. Furthermore, a strong positivity of Snail, vimentin, and collagen type I in pSS specimens was detected as compared with normal SG tissue, while, at the same time, the expression levels of E-cadherin were decreased in diseased SG biopsies. All this evidence suggests that TGF-β1 is able to activate the EMT program in SGEC through the canonical TGF-β1/SMAD/Snail pathway [17,25,35] (Figure 4).

Recently, research in this field has elicited innovative results showing that, in the markedly inflammatory microenvironment of pSS SGs, characterized by pro-inflammatory cytokines release by glandular epithelial cells and infiltrating lymphocytes, the loss of epithelial markers such as E-cadherin, and the acquisition of mesenchymal markers such as vimentin and collagen type I, increased in line with the grade of inflammation. In particular, specific pro-inflammatory cytokines such as IL-17 and IL-22 participate in TGF-β1/EMT-dependent SGs fibrosis [235], confirming data obtained in other experimental studies performed on several tissues. It is now accepted, for example, that IL-17 triggers EMT in airway epithelial cells dependent on TGF-β1 [236,237]. Moreover, IL-22 determines a dramatic up-regulation of TGF-β1, α-SMA, laminin, and hyaluronic acid and collagen type IV gene expression and transcription in human hepatic stellate cells, promoting severe liver fibrosis [238]. High levels of IL-22, strictly associated with hyposalivation, have been shown in pSS patients’ sera [239] and, in pSS biopsies, IL-22 and IL-17 are abundantly present in the inflamed SGs, and correlated with the degree of tissue inflammation [239,240].

Interestingly, during IL-17 and IL-22 experimental treatment of healthy SGEC, these cells undergo a remodelling of their morphological characteristics, assuming a spindle-like mesenchymal phenotype, which is indicative of the beginning of an EMT process. In particular, during an experimental study performed on healthy SGEC using IL-17 as stimulus, healthy SGEC underwent the phosphorylation and activation of Smad2/3 and Erk1/2 and EMT. Interestingly, the activation of the canonical TGF-β1/Smad2/3 and non-canonical TGF-β1/Erk1/2 pathway in IL-17-treated healthy SGEC was demonstrated. In addition, it is very important to note that the IL-17-dependent/TGFβ1-induced EMT was detected in human healthy SGEC. The validity of these data was confirmed by co-treatment of the SGEC with IL-17 and specific TGFβ receptor type I kinase or Erk 1/2 inhibitors that abrogate EMT activation in healthy SGEC. Importantly, another piece of the puzzle in understanding the molecular mechanisms underlying EMT activation has been added, showing that the inhibition of canonical TGFβ1/Smad2/3 signalling transduction has no effect on the activation of the non-canonical TGFβ1/Erk1/2/EMT pathway. This suggests that at least two pathways contribute, canonically or not, to activating the IL-17-dependent EMT process in SGEC [25,241]. The same results were obtained using IL-6 as pro-fibrotic factor, which determined the activation of EMT in a dose-dependent manner in healthy SGEC [235]. IL-6 treatment induces a decreased gene and protein expression of E-cadherin, accompanied by increased levels of vimentin and collagen type I. This corroborates the hypothesis that a dysregulated pro-inflammatory factors production during the chronic inflammation characterizing pSS SGs may contribute to the onset and progression of EMT-dependent fibrosis in pSS [25,241] (Figure 4).

## 11. Autoimmune Hepatitis and Liver Fibrosis

Autoimmune hepatitis (AIH) is a chronic inflammatory disorder of unknown aetiology, characterized by periportal inflammation, elevated circulating autoantibodies, and a sustained fibrogenesis characterized by an alteration of the liver parenchyma and vascular architecture. Fibrosis in AIH is a consequence of chronicity of liver damage, a condition that is diagnosed during flares of disease [242]. Although the onset of fibrotic events in the AIH is poor unknown, over the last decade, strong evidences point out the relevant role of the TGF-β signalling during all phases of the development of liver fibrosis. In preliminary studies, Bayer et al. demonstrated a strong TGF-β expression in the inflamed liver and, in particular, many hepatocytes showed strong staining for TGF-β. These results suggested that TGF-β may be an important mediator in active autoimmune hepatitis [243]. Moreover, a key discovery in understanding liver fibrosis has been that, not only the hepatocytes, but also the non-parenchymal cells such as hepatic stellate cells (HSCs) are essential for maintaining an intact liver structure and function. This cell type undergoes a severe phenotypic modification in chronic liver diseases with the acquisition of fibrogenic characteristics, playing a central role in the development of liver fibrosis [244]. Recently, emerging evidence suggests that the activation of the EMT program concurs to HSC differentiation in myofibroblasts-like cells [245]. This phenomenon can be caused by a range of chronic injuries to the liver, amongst which there is autoimmune hepatitis [242]. Therefore, hepatocytes are also able to undergo EMT in response to liver damage during the progression of chronic autoimmune hepatitis. Even more, primary hepatocytes could differentiate into a fibroblastic-like phenotype losing cellular contacts and polarity, after TGF-β stimulation [246]. Indeed, fibroblasts derived from hepatocytes are an additional and significant lineage of mesenchymal cells that concur to development of liver fibrosis. Zeisberg and collaborators demonstrated that adult hepatocytes can undergo an EMT process after treatment of TGF-β, determining a pool of fibroblast during liver fibrosis [16]. Moreover, Rowe et al. have showed that hepatocytes modulate Snail protein expression during liver tissue remodeling. The ablation of Snail in hepatocytes in vivo, demonstrates that this transcription factor plays a pivotal role in the development of liver fibrosis by inducing several events of fibrogenesis, including growth factor expression, ECM accumulation, and chronic inflammatory responses [247].

## 12. Conclusions

There is strong evidence of the EMT role in fibrosis, associated to chronic inflammatory conditions and autoimmunity but further observations will be needed to determine whether a full trans-differentiation of epithelial cells to myofibroblasts does really occur in vivo. Whether an EMT program activation occurs may depend on the stimulus used in the laboratory or on the organ in which fibrosis is triggered, and there may be differences between in vitro cellular experiments and human diseases. Decodifying the fibroblast phenotype by molecular profiling in various autoimmune fibrotic disorders may identify distinct fibroblast populations in the different target organs involved in the pathogenesis of autoimmune diseases. This could offer insights helping to clarify whether these populations derived from different cellular sources. Although preliminary in some cases, there is now evidence that epithelial cells can undergo a transition to a mesenchymal phenotype through EMT and directly promote organs fibrosis. A better understanding of the precise molecular interactions that lead to EMT will hopefully advance our understanding and ability to treat fibrosis and resulting disease processes. Preclinical studies have claimed TGF-β signalling molecules as promising anti-fibrotic drug targets in various organ fibrosis animal models, whereas a vast majority of clinical studies have not yet achieved an acceptable level of therapeutic performance. The broad distribution and pleiotropic expression of TGF-β signaling in physiological tissue is the major hurdles in targeting the TGF-β pathway for fibrosis treatment. Therefore, TGFβ signalling inhibitors, actually used, are generally safe and may be efficacious in several clinical applications, especially in severe cases such as end-stage cancer. This may suggest that the inhibition of the TGFβ signalling pathway may be beneficial also in inflammatory autoimmune disorders, but future investigations are needed to prove this hypothesis.
